# A Novel Multi-Task Learning Network Based on Melanoma Segmentation and Classification with Skin Lesion Images

**DOI:** 10.3390/diagnostics13020262

**Published:** 2023-01-10

**Authors:** Fayadh Alenezi, Ammar Armghan, Kemal Polat

**Affiliations:** 1 Department of Electrical Engineering, College of Engineering, Jouf University, Sakaka 72388, Saudi Arabia; 2 Department of Electrical and Electronics Engineering, Bolu Abant Izzet Baysal University, Bolu 14280, Turkey

**Keywords:** melanoma classification and segmentation, deep learning, super-resolution, multi-task learning network

## Abstract

Melanoma is known worldwide as a malignant tumor and the fastest-growing skin cancer type. It is a very life-threatening disease with a high mortality rate. Automatic melanoma detection improves the early detection of the disease and the survival rate. In accordance with this purpose, we presented a multi-task learning approach based on melanoma recognition with dermoscopy images. Firstly, an effective pre-processing approach based on max pooling, contrast, and shape filters is used to eliminate hair details and to perform image enhancement operations. Next, the lesion region was segmented with a VGGNet model-based FCN Layer architecture using enhanced images. Later, a cropping process was performed for the detected lesions. Then, the cropped images were converted to the input size of the classifier model using the very deep super-resolution neural network approach, and the decrease in image resolution was minimized. Finally, a deep learning network approach based on pre-trained convolutional neural networks was developed for melanoma classification. We used the International Skin Imaging Collaboration, a publicly available dermoscopic skin lesion dataset in experimental studies. While the performance measures of accuracy, specificity, precision, and sensitivity, obtained for segmentation of the lesion region, were produced at rates of 96.99%, 92.53%, 97.65%, and 98.41%, respectively, the performance measures achieved rates for classification of 97.73%, 99.83%, 99.83%, and 95.67%, respectively.

## 1. Introduction

Skin cancer has a higher incidence compared to other types of cancer. There are two types of skin cancer: melanoma and non-melanoma. The uncontrolled growth of pigmented cells (melanocytes) causes melanoma. Skin cancer deaths from melanoma have stably increased each year over the past years [1,2]. Based on these data, it can be said that this increase poses a significant threat to public health. Early detection is important in order to save human life. Melanoma skin cancer has a successful cure rate when detected early. For this reason, the importance of the methods used in the early detection of the disease has increased. The distinction between lesioned and non-lesioned areas on melanoma skin cancer images is complex in normal conditions. It is not easy to distinguish between these areas, a task that requires specialty.

For this reason, it may cause differences of opinion among dermatologists. It is argued that in order to solve this problem and for dermatologists to be able to make both an accurate and rapid diagnosis, an automated analysis system is needed [3,4,5,6]. Automatic segmentation of the skin surrounding melanomas is an essential step in the computerized analysis of the dermoscopic image [7].

As today’s technology develops, the prevalence of deep neural networks has increased rapidly. Convolutional neural network (CNN) architectures, usually chosen in computer vision applications, possess feature extraction and classification abilities based on deep learning [8,9,10,11]. In recent years, high-performance results have been acquired for pattern recognition and segmentation with CNNs [12,13,14,15,16]. In this vein, many studies have been carried out in the segmentation and classification of lesions occurring on the skin. In these studies [17,18,19,20,21,22,23,24], pre-trained CNN models based on the transfer learning were used to classify the skin lesions. On the other hand, pixel essential lesion regions were generally detected by deep learning approaches such as UNet [25,26,27,28,29,30,31], Mask R-CNN [32,33], fully convolutional networks [34,35], feature pyramid networks [36], SegNet [37,38], and transformers [39,40]. 

As in the current study, many studies based on the segmentation and classification processes were carried out to identify skin lesions with dermoscopy images. These studies are primarily based on the segmentation of lesions from dermoscopy images and the subsequent classification of these detected lesions. Seeja and Suresh (2019) proposed a mixed model for melanoma classification and segmentation with dermoscopic images from these studies. They first detected lesions with the UNet model and then extracted the shape, color, and texture features from segmented images. Finally, naive Bayes, SVM, and k-nearest neighbor machine learning classifiers were used for melanoma classification using these features. These results provided 85.19% accuracy in classifying nelanoma and a 77.5% Dice co-efficiency value for lesion segmentation [41]. Ding et al. (2022) proposed a two-stage deep neural network. This model applied five deep architectures to lesion images detected from UNet architecture. They achieved 90.9% accuracy with the ISIC 2017 dataset [42]. Jojoa Acosta et al. (2021) first cropped the lesions from the skin image using Mask R_CNN architecture. Then, they used the pre-trained ResNet152 architecture to classify the cropped lesions. This model produced 90.4% accuracy with the ISIC 2017 dataset [43]. Malibari et al. (2022) presented a deep model, based on skin classification and detection with a deep convolutional neural network, in another study. When pre-trained Squeezenet architecture-based whale optimization was used for the classification step, they utilized the UNET architecture for the segmentation step. As a result, they reached 99% accuracy with the ISIC 2019 dataset for melanoma classification [44]. Jayapriya and Jacob (2020) used a hybrid structure with a deep convolutional network. They proposed a method to extract features from the lesions, segmented using pre-trained fully convolutional networks, and classify them with SVM. Experimental works observed that this hybrid approach produced an accuracy of 85.3% for ISIC 2017 and 88.92% for ISIC 2016 [45].

This paper proposes a multi-task learning network based on the melanoma segmentation and classification of skin lesion images. In the segmentation phase of the proposed model, pre-processing methods were used. First, the hair details in the image were removed, and the image was clarified. Later, the lesions in the images were segmented using the VGGNet model-based FCNLayer architecture. Finally, the cropping of these lesions was performed, and the image resolution was increased using a very deep super-resolution neural network. These high-resolution images obtained were given to the input of the classifier model. The classifier model presents a new approach based on three powerful pre-trained deep models. ISIC database was used to test the performance of the proposed approaches. The experimental results show that the multi-task learning network model developed based on segmentation and classification provides high performance.

The contribution of the proposed deep network approach are as follows:

- Lesion images, cropped from the images detected in the segmentation process, were converted to the input size of the classifier model using the very deep super-resolution neural network approach, and the resolution of lesion images was raised.
- Lesions were correctly located in all dermoscopy images with the VGGNet-based FCNLayers approach. The numerical and visual results obtained from experimental studies proved this situation.
- In this paper, an effective deep network architecture is proposed, based on the combination of deep models with different structures. In experimental studies, the proposed approach has been observed to achieve outstanding success in classifying melanoma.

The remainder of the study proceeded as follows. The methodology and theoretical framework are given in Section 2. Experiment results and information about the data set are announced in Section 3. In Section 4, the results are of the model presented when using the developing new technologies, and the study is generally concluded with Section 4.

## 2. Materials and Methods

**Figure 1:**
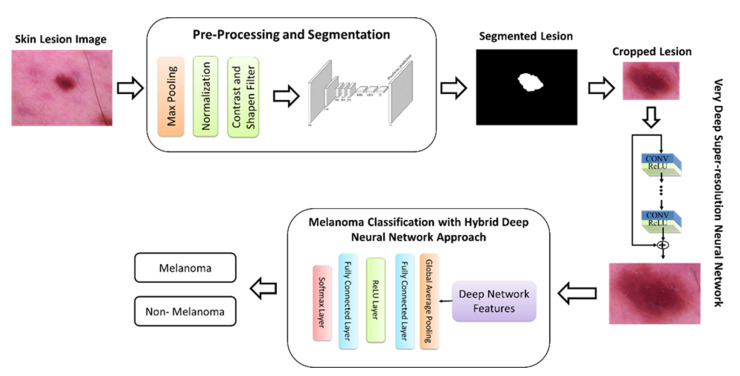
The proposed multi-task learning network.

In the current study, we presented a paper, as well as a multi-task learning network based on melanoma recognition with dermoscopy images. The proposed system consists of two main stages: segmentation and classification. In the segmentation phase, operations such as the removal of hair details, detection, and cropping of the lesion region are included. The classification phase includes obtaining high-resolution images and classifying melanoma based on the deep neural network. The general representation of the proposed system, which includes all these processes, is given in Figure 1.

### 2.1. Segmentation

**Figure 2:**
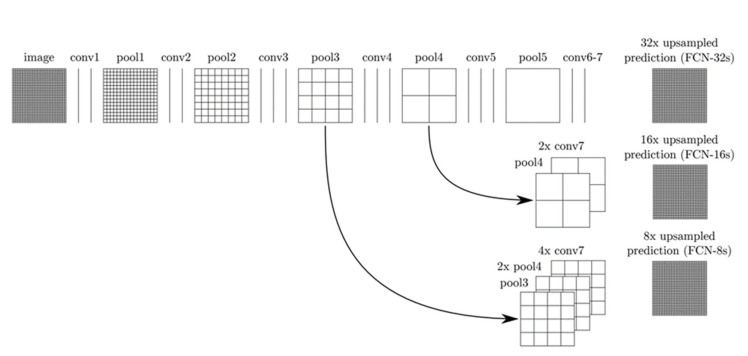
The structure of FCNLayer architecture [46].

In this study, a deep learning approach, based on the segmentation of high-resolution images, has been advanced. This approach, using 2 stages, consists of pre-processing and detection. In the “pre-processing” approach, the segmentation includes image enhancement operations to improve the forecasting results. Firstly, maximum pooling, contrast, and sharpening methods were applied to remove hair details from skin lesions images and to clarify the image. Then, the VGGNet-based FCNLayer approach was used to detect the lesion region from the enhanced dermoscopy images. This architecture [46] is based on a pixel-based fully convolutional network semantic segmentation. The general structure of this architecture is given in Figure 2.

In the structure given in Figure 2, the gridded rectangles represent the pooling and prediction layers, while the vertical lines represent the interlayers. There are 3 models that are based on the FCNLayer architecture. These are known as 8 s, 16 s, and 32 s. In the first row in Figure 2, the FCN-32s return the upsampling of the 32 predictions back to the images in a single step. In the second line, the FCN-16s divide the output in two by 16 pixels. A 1 × 1 convolution layer is added to the fourth pooling layer to create additional forecasts. Then, the ×2 upsampling layer is added and combined with the forecasts computed in the seventh convolution layer. This eturns upsamples of 16 forecasts back to images with ×2 multiples. FCN-8s are on the third line; FCN-16 is ×2, and by adding a 1 × 1 matrix to the third pooling layer, it is combined with the predictions computed in the seventh convolution layer and returns upsamples of 8 forecasts back to images with ×4 multiples [46]. 

**Figure 3:**
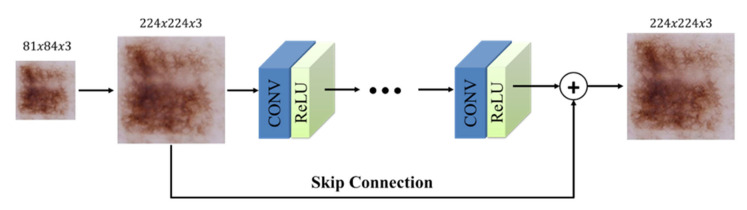
The structure of VSDR architecture [47].

In the current study, the detection of the lesion area in skin images was realized using VGGNet-based FCN-32s, FCN-16s, and FCN-8s approaches. Then, clipping was performed for the detected lesions. Finally, there was a need to increase the size of the cropped lesion images to be given to the input of the classifier model. This operation converts images to the desired size using the interpolation method. However, changing the size of the image with this method also affects its resolution. Accordingly, we used a deep learning approach based on a very deep super-resolution neural network [47]. This architecture aims to raise the image quality by re-inserting the lost details into the image. This architecture provides increased model performance by combining low-level features and high-level features through a skip link [48]. The VSDR architecture consists of cascading convolutional layers with a size of 3 × 3 × 64, and the size of the part of the image in question is 41 by 41. The general structure of this network architecture is given in Figure 3.

**Figure 4:**
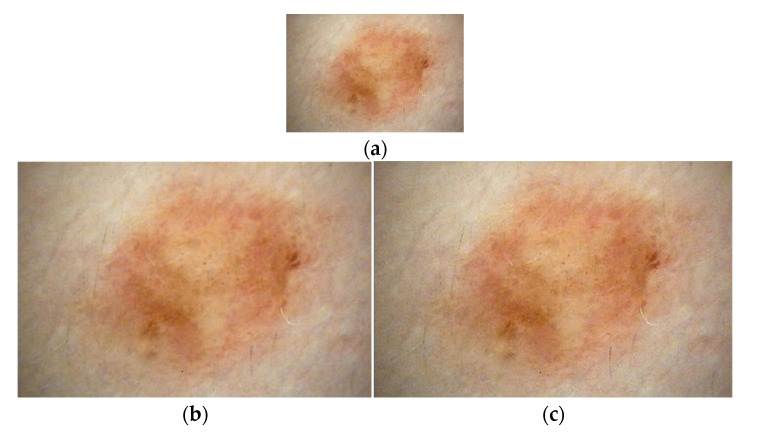
Size enhancement samples (**a**) Original image, (**b**) The bilinear interpolation, (**c**) The proposed VSDR approach.

An example illustration of this process is given in Figure 4. In this example, the 48 × 64 cropped lesion image was converted to a 224 × 224 size by applying bilinear interpolation (Figure 4b) and the proposed approach (Figure 4c). As a result, it was observed that the image resolution was better with the proposed VSDR approach.

### 2.2. Classification

**Figure 5:**
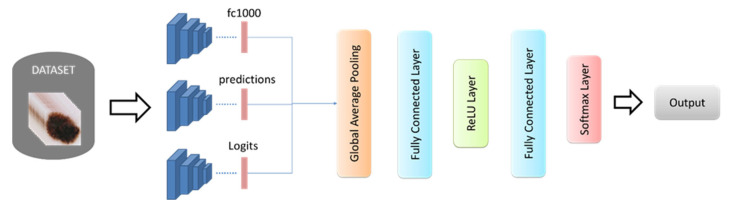
The structure of the proposed classifier model.

This paper proposed a deep approach based on image super-resolutions and multiple pre-trained convolutional neural networks to classify skin lesions. The general flow diagram of the proposed model is given in Figure 5.

In the classification model, the learned weights of the pre-trained deep architectures are used instead of re-developing and training a CNN model from scratch [49,50,51,52,53,54,55,56]. For this purpose, deep architectures such as DenseNet, GoogleNet, and MobileNet with high performance were used. These architectures have different structures from each other. Detailed information about these architectures is given below:

- DenseNet201: The DenseNet model is a network architecture in that every layer forwards directly links up other layers [57]. This architecture can reuse the features of different layers, which allows for an increase in the diversity of the input of the next layer and improves performance [58]. It also provides for a direct connection between any two layers with the same graph size and allows the network features to be regained in learning the model [59]. Each layer’s feature maps are passed as inputs to all subsequent layers, while the feature maps of all former layers are approached as apart inputs. Besides, in the DenseNet model, the pooling layer and bottleneck mold are used for transition layers to make feature parameters more efficient and reduce methodological complexity [60,61]. ResNet and DenseNet architectures have similarg architectures. However, in ResNet architecture, every ResNet model receives knowledge from the former model, while in DenseNet architecture, each layer resides receiving knowledge from former layers. The divergence in the DenseNet model joins each layer in a feed-forward intensely [60].
- GoogleNet: This network was developed in 2015 as a broader and deeper CNN model [62]. GoogleNet has inception modules (1 × 1, 3 × 3, and 5 × 5 convolution sublayers) that perform different sizes of folds and combine filters for the next layer. It has a maximum pooling layer of 3 × 3, capable of performing parallel operations [63,64]. These layers acquire data from former layers and then perform these parallel operations. To reduce the losses in the computation, a 1 × 1 convolution is performed before these operations, but in the beginning module, the 1 × 1 sub-convolution layer is placed after the maximum pooling layer. In every part of the beginning layer, features that may differ from the previous data are calculated. Every output is then combined as an input for the other layers of this CNN. This model uses starter modules instead of fully connected layers. Maximum pooling between some layers is carried out in this network to reduce the information coming from important layers. As well, in GoogleNet, an average pooling layer is available at the end of the network [64,65,66].
- MobileNetv2: This network implements a technique called deeply separable convolutions (DSC) and uses linear bottlenecks to enhance the information extinction problem that occurs in nonlinear layers in convolution blocks [67,68]. It also introduces a new structure, called inverse residuals, to preserve information. The MobileNet architecture is based on deep, separable convolution. All input channels are processed along the standard convolution and inverted along the depth then convolution of all the inputs with the filter channel. Thus, an output channel with a filter is obtained. These channels are then stacked. Deep convolution uses 1 × 1 convolution to combine these channels into a single channel. As a result, it is known that although this method produces the same outputs as standard convolution, it reduces the number of parameters and increases efficiency [67,69].

In the proposed approach, first, fully connected layers of these architectures were used and 1000 deep features were extracted from dermoscopy images for each. The pseudocode based on these FC layers is given in Equation (1).

(1) $$feat\_Dense_{k}=activation\left(DenseNet_{pretrainedparameters},image_{k}{,}^{\prime }fc1000^{\prime }\right)\;feat\_Google_{k}=activation\left(GoogleNet_{pretrainedparameters},image_{k}{,}^{\prime }loss3-classifier\prime \right)\;feat\_Mobile_{k}=activation\left(MobileNet_{pretrainedparameters},image_{k}{,}^{\prime }Logits\prime \right)\;k=1,2,3,\ldots ,N$$

where, *N* represents the number of images in the dataset. The deep features obtained using Equation (1) were combined using the global average pooling layer, and 1000 features were obtained for each image Equation (2).

(2) $$feat_{k}=\overset{N}{\underset{k=1}{\sum }}\frac{1}{3}\left(\overset{1000}{\underset{i=1}{\sum }}feat\_Dense_{i}+feat\_Google_{i}+feat\_Mobile_{i}\right)$$

Finally, *N* × 1000 feature vectors are given as input for a feature layer. This layer is followed by fully connected, ReLU, fully connected, and softmax layers, respectively. As a result, the training process was carried out using the developed deep learning network’s architecture.

## 3. Results

In the current study, we presented a multi-task learning network based on melanoma segmentation and classification with dermoscopy images. In experimental works, the confusion matrix was previously used to calculate the performance of the proposed segmentation and classification model.

In the experimental studies carried out, the test and training sets for two data sets were randomly divided as 20% and 80%, respectively, to realize one time only. In this way, using the same test and training data set for all applications, the effects of indiscriminately divided data on performance were minimized.

### 3.1. Dataset

**Figure 6:**
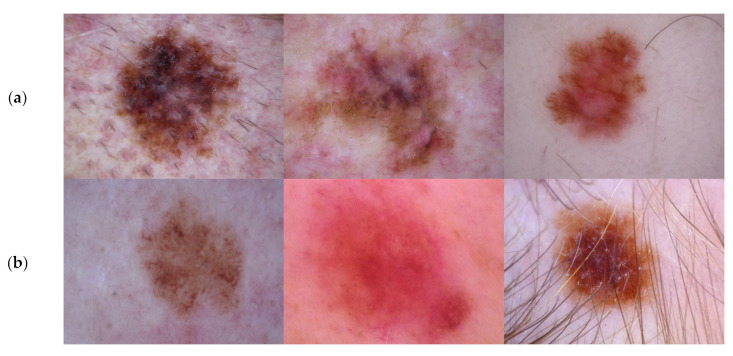
Sample dermoscopy images, (**a**) Melanoma, (**b**) Non-melanoma.

In experimental studies, the widely used, publicly available HAM10000 dataset was used to evaluate the performance of the proposed classification and segmentation models. This dataset consists of a total of 10,015 dermoscopic images belonging to seven classes: benign keratosis, melanoma, basal cell carcinoma, vascular lesion, dermatofibroma, melanocytic nevi, and actinic keratosis, as shown in Figure 6. Additionally, it is an unbalanced dataset, containing a different number of images for each class. We performed experimental studies for two classes, 1113 melanoma images and 8902 non-melanoma images in the current study. A data imbalance between these two classes can lead to overfitting during the training phase. Therefore, we used the data augmentation method, rotation, flip, contrast, and bright, to equalize the data numbers. In this process, the separation of training and test data was performed on the raw data set, and the data was balanced by using data augmentation methods for these two separate datasets. After these processes, the number of melanoma images was increased by 8904 and thus obtained the dataset a total of 17,806 images. Figure 6 shows sample dermoscopy images, (a) Melanoma, (b) Non-melanoma.

### 3.2. Result of Skin Lesion Segmentation

In the experimental study, we used VGGNet-FCN-8s, VGGNet-FCN-16s, and VGGNet-FCN-32s models, based on the pre-trained VGG16 architecture, for lesion segmentation. In this experimental study, deep parameters such as epoch size 200, batch size 1, and Adam optimization method were used in training these approaches. The TP, FP, TP, and TN values obtained, based on the confusion matrix for each model, are given in Table 1.

The performance measures such as accuracy, precision, and sensitivity were calculated according to the confusion matrices obtained from the models given in Table 1 and are given in Table 2.

**Figure 7:**
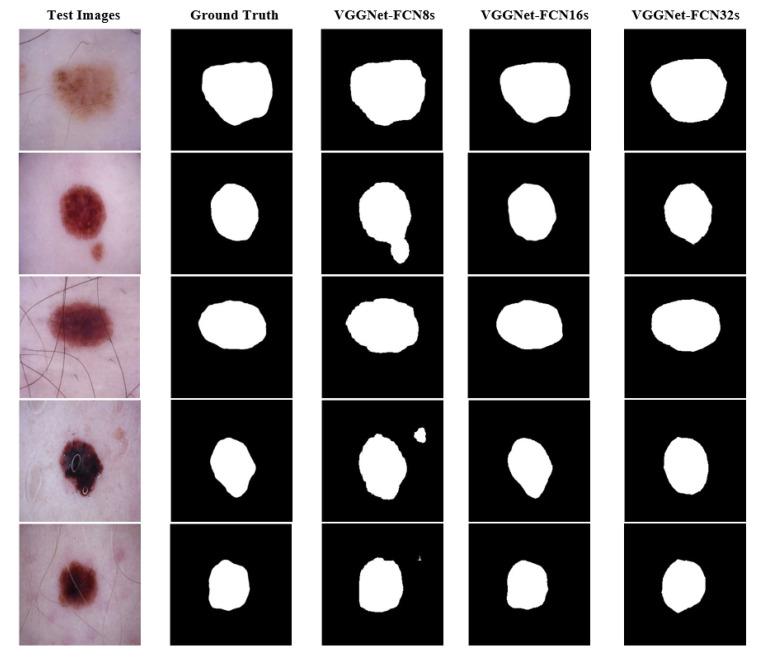
The visual estimation results based on sample dermoscopy images.

According to the results given in Table 2, it was observed that the best performance among the proposed approaches was obtained with VGGNet-FCN16s. On the other hand, the VGGNet-FCN32s model produced 96.11% accuracy, 96.71% precision, and 98.17% sensitivity values, while VGGNet-FCN8s produced 93.61%, 92.59%, and 98.99%, respectively. In addition, sample visual prediction results, based on VGGNet-FCN approaches, are given in Figure 7.

According to the visual estimation results given in Figure 7, it is clearly observed that the VGGNet-FCN16s approach is more successful than other approaches. In addition, while the VGGNet-FCN8s model correctly detected the locations of lesion regions, it also detected non-lesion regions as lesions.

### 3.3. Result of Skin Lesion Classification

In this classification stage, the individual performances of pre-trained deep architectures such as DenseNet, MobileNet, and GoogleNet, based on the transfer learning approach, were calculated using the cropped images obtained from the segmentation process. These results are given in Table 3. The image size, given for the input of each deep architecture, was adjusted using the VSDR network approach, and a possible resolution reduction was prevented. In these experimental studies, the epoch size, batch size, and optimization method were set to 100, 32, and Sgdm (stochastic gradient descent with momentum), respectively. 

**Figure 8:**
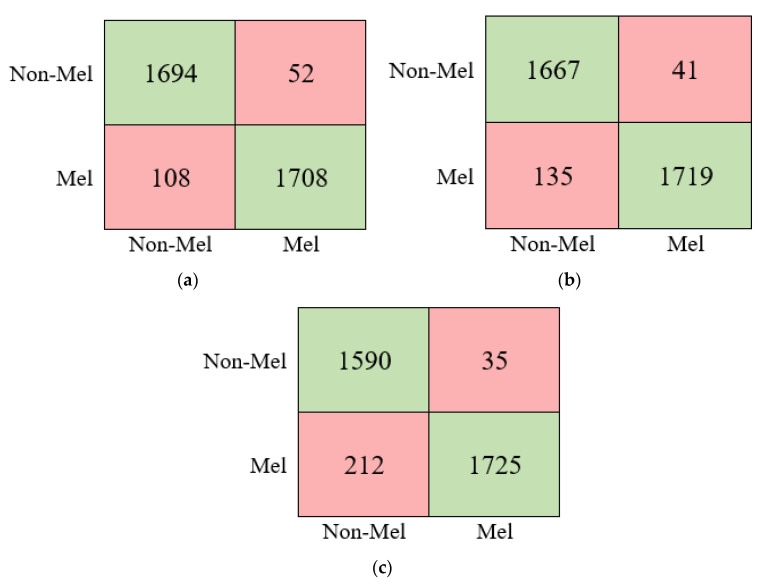
The confusion matrices of pre-trained deep models, (**a**) DenseNet, (**b**) MobileNet, (**c**) GoogleNet.

According to the results given in Table 3, the best performance among the deep models was obtained with DenseNet at 95.51%. In addition, the MobileNet and GoogleNet models produced 95.06% and 93.07% accuracy, respectively. The confusion matrices of these models are given in Figure 8. 

Finally, deep architectures with three different structures used in this study were combined according to the proposed classifier model given in Figure 2. The performances of the combinations were calculated. The obtained performance values are given in Table 4. 

**Figure 9:**
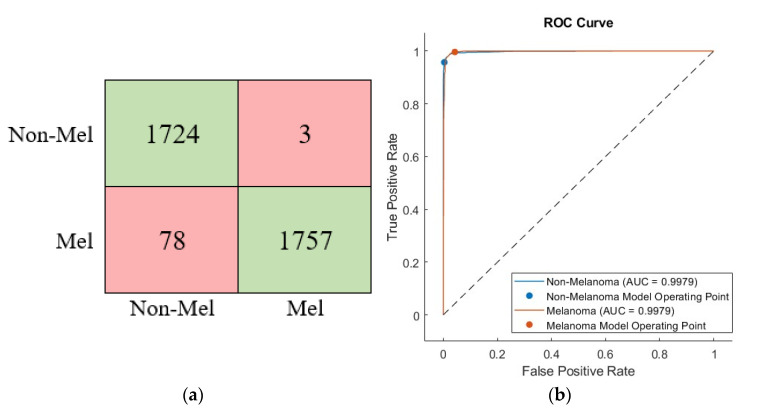
The confusion matrix (**a**) and ROC diagram (**b**) of proposed classifier approach.

As can be seen from Table 4, the deep learning network approach developed based on the three deep models achieved the best accuracy of 97.73%. On the other hand, the second-best score was obtained by combining the Mobilenet and Densenet architectures. In addition, the confusion matrix and ROC diagram of the proposed approach (D+G+M) are given in Figure 9. 

## 4. Discussion

Recently, considering the outstanding achievements of deep learning algorithms, many studies have been carried out in the last 3–4 years for the segmentation and classification of melanoma. In these studies, segmentation and classification processes, based on deep convolution neural networks, were performed using ISIC datasets in general. The performances of the previous studies based on the HAM1000 dataset are compared with the proposed model, and these results are given in Table 5.

When the previous studies given in Table 5 are examined, either classification or segmentation studies were generally performed based on dermoscopy images. Deep learning models such as UNet, FCN, and Segnet were generally used in studies based on the segmentation process. As seen in the results obtained in the experimental findings of the current study, it was observed that the FCNLayer architecture produced more successful results than the other models developed. On the other hand, it is known that networks, trained from scratch based on convolutional neural networks, provide lower levels of performance than pre-trained deep architectures. Therefore, pre-trained deep models were used in most studies based on skin lesion classification. In the current study, it was preferred to use pre-trained deep models based on the transfer learning approach. Accordingly, the developed hybrid deep learning network model was more successful than that in other studies, with an accuracy score of 97.73%. 

This paper presents a multi-task learning network, covering segmentation and classification processes. There are a few studies similar to the proposed approach, such as those of Khan et al. (2021) [78] and Khan et al. (2021) [79]. In these studies, when the results obtained for both processes were examined, it was clearly observed that superior performance was obtained compared to previous studies. In these studies, when the results obtained for both processes were examined, it was clearly observed that superior performance was obtained with the proposed approach. 

## 5. Conclusions

The current study proposed a novel approach, based on a multi-task learning network, for melanoma recognition with dermoscopy images. This model includes a hybrid approach based on segmentation and classification. In the segmentation phase, hair details in dermoscopy images were removed, and lesion regions were detected with the VGGNEt-based FCNLayers approach. The experimental results obtained high performances, with 97.65% precision and 98.41% sensitivity scores. In addition, when the visual estimation results were examined, it was observed that the developed approach correctly detected the positions of the lesions in all images. On the other hand, lesion images cropped from the images detected in the segmentation process were converted to the input size of the classifier model using the very deep super-resolution neural network approach, and the resolution of the lesion images was raised. Then, the proposed classifier model, based on three powerful pre-trained deep architectures with different structures, was tested using the ISIC dataset. The experimental results displayed high performance with an accuracy score of approximately 97.73%. As a result, deep learning network approaches, proposed for segmentation and classification processes, were observed to be more successful than they were in previous studies.

In future studies, we will focus on optimization methods for the variables used in the proposed approach and parameters that affect performance. In addition, the transformer structure will be examined, and it will be considered to adapted the proposed approach.

## Figures and Tables

**Table 1:** Confusion matrix values (Pixel) of the proposed approach.

	TP	FP	FN	TN
VGGNet-FCN8s	45,969,975	3,681,826	466,798	14,797,339
VGGNet-FCN16s	48,483,463	1,168,338	782,699	14,481,438
VGGNet-FCN32s	48,019,878	1,631,923	896,535	14,367,602

**Table 2:** The performance results (%) of VGGNet-FCN-based approaches.

	Accuracy	Precision	Sensitivity
VGGNet-FCN8s	93.61	92.59	98.99
VGGNet-FCN16s	96.99	97.65	98.41
VGGNet-FCN32s	96.11	96.71	98.17

**Table 3:** Classification results (%) of pre-trained deep models.

	Accuracy	Specificity	Precision	Sensitivity
DenseNet	95.51	97.05	97.02	94.01
MobileNet	95.06	97.67	97.60	92.51
GoogleNet	93.07	98.01	97.85	88.24

**Table 4:** Classification results (%) of proposed deep approaches (DenseNet: D, GoogleNet: G, MobileNet: M).

	Accuracy	Specificity	Precision	Sensitivity
D+G	95.84	99.61	99.56	91.32
G+M	96.35	98.45	98.33	94.17
M+D	97.16	99.78	99.76	94.46
D+G+M (our)	97.73	99.83	99.83	95.67

**Table 5:** Comparison of the proposed model’s segmentation and classification results (%) with previous studies.

References	Task	Accuracy	Specificity	Precision	Sensitivity
Alam et al. (2022) [70]	Classification	91	-	-	-
Srinivasu et al. (2021) [71]		90.21	95.1	-	92.24
Dhivyaa et al. (2020) [72]		97.3	-	-	-
Bibi et al. (2022) [73]		96.7		94.48	-
Barın and Güraksın (2022) [74]	Segmentation	94.65	87.86		95.85
Wu et al. (2022) [40]		95.78	96.99		91
Jin et al. (2021) [75]		93.4	90.4		96.7
Lei et al. (2020) [76]		92.9	91.1		95.3
Hussain and Basak (2021) [77]		-	93.8	-	94.3
Khan et al. (2021) [78]	Segmentation	92.69	-	-	-
	Classification	90.67	-	-	90.2
Khan et al. (2021) [79]	Segmentation	92.25	-	-	-
	Classification	88.39	-	-	-
Our method (2022)	Segmentation	96.99	92.53	97.65	98.41
	Classification	97.73	99.83	99.83	95.67

## Data Availability

The introduced public datasets are available: HAM10000 dataset at https://www.kaggle.com/datasets/kmader/skin-cancer-mnist-ham10000 (accessed on 1 August 2022).
